# Using a Human Papillomavirus Model to Study DNA Replication and Repair of Wild Type and Damaged DNA Templates in Mammalian Cells

**DOI:** 10.3390/ijms21207564

**Published:** 2020-10-13

**Authors:** Dipon Das, Molly L. Bristol, Pietro Pichierri, Iain M. Morgan

**Affiliations:** 1 Department of Oral and Craniofacial Molecular Biology, VCU Philips Institute for Oral Health Research, Virginia Commonwealth University School of Dentistry, Richmond, VA 23298, USA; dipon.das@hotmail.com (D.D.); mlbristol@vcu.edu (M.L.B.); 2 Department of Environment and Health, Istituto Superiore di Sanita’, 00161 Rome, Italy; pietro.pichierri@iss.it; 3 VCU Massey Cancer Center, Richmond, VA 23298, USA

**Keywords:** human papillomaviruses, DNA replication, replication and repair, DNA damage, model system, E1 and E2, cervical cancer, head and neck cancer, DNA lesion

## Abstract

Human papillomaviruses have 8kbp DNA episomal genomes that replicate autonomously from host DNA. During initial infection, the virus increases its copy number to 20–50 copies per cell, causing torsional stress on the replicating DNA. This activates the DNA damage response (DDR) and HPV replicates its genome, at least in part, using homologous recombination. An active DDR is on throughout the HPV life cycle. Two viral proteins are required for replication of the viral genome; E2 binds to 12bp palindromic sequences around the A/T rich origin of replication and recruits the viral helicase E1 via a protein–protein interaction. E1 forms a di-hexameric complex that replicates the viral genome in association with host factors. Transient replication assays following transfection with E1–E2 expression plasmids, along with an origin containing plasmid, allow monitoring of E1-E2 replication activity. Incorporating a bacterial lacZ gene into the origin plasmid allows for the determination of replication fidelity. Here we describe how we exploited this system to investigate replication and repair in mammalian cells, including using damaged DNA templates. We propose that this system has the potential to enhance the understanding of cellular components involved in DNA replication and repair.

## 1. Introduction

Human papillomaviruses (HPV) are causative agents in around 5% of all cancers, including the majority of cervical and oropharyngeal cancers [1]. HPV are separated into low risk (LR-HPV, those that do not cause cancer) and high risk (HR-HPV, those that do cause cancer) types; HPV16 is the most prevalent HR-HPV and is responsible for around 50% of cervical cancers and 90% of HPV-positive oropharyngeal cancers. HPV also cause anal, penile, and vulvar cancer [2,3]. HPV are epitheliotropic; this review will only discuss HR-HPV.

HPV infect the basal cells of the epithelium following abrasions that allow access [4]. Via a complicated number of steps, the viral DNA enters the host nuclei following mitosis [5]. Host factors then bind to the viral transcriptional control region (the long control region, LCR) and activate transcription from the viral genome [6]. The viral transcript is processed to produce RNA species that are translated into individual viral proteins. We will give a very brief review of the viral transforming proteins so that the reader can understand how HPV causes cancer. E6 binds to p53 and targets it for proteasomal degradation via an interaction with E6AP, a HECT E3 ubiquitin ligase; E6 also targets a host of other proteins to regulate their function [7,8,9]. E7 binds to pRb and disrupts its interaction with E2F1, resulting in the activation of S-phase genes via the relief of pRb repression of the E2F1 transcriptional activation function [10,11,12,13,14]. E7 also targets pRb for degradation and targets additional pocket proteins, including p107 and p130. E7 also regulates the differentiation of the infected epithelium. The disruption of the latter complexes disrupts the dimerization partner, RB-like, E2F and multi-vulval class B (DREAM) complex, resulting in the activation of proliferative genes [15]. The overall result of E6 and E7 expression is the stimulation of the cell cycle (driven by E7) and a failure by the cell to recognize this aberrant proliferation (driven by E6). E5 is also involved in the induction of proliferation of the infected cell, although its precise contributions remain to be fully elucidated [16]. E5, E6, and E7 are the viral oncogenes expressed by HR-HPV, and all HPV tumors retain the expression of E6/E7, which are drivers of the transformed phenotype.

There are two viral proteins required for replication. The E2 protein forms a homodimer via a carboxyl terminus dimerization domain and binds to four 12bp palindromic sequences on the LCR [17]. Three of these binding sites surround the A/T rich origin of viral replication. The E1 protein is a helicase [18]. It is recruited to the A/T rich origin of replication in the LCR via a protein–protein interaction with the E2 protein amino terminal domain [19,20]. Following recruitment, E1 forms a di-hexameric helicase complex that interacts with host polymerases and factors to initiate and complete replication of the viral genome [18]. 

Following initial infection and expression of the viral proteins, the viral genome copy number increases to 20–50 copies per cell to establish an infection [21,22]. The infected basal cell is induced to proliferate via the action of the viral oncogenes and the infected cell migrates “upwards” in the infected epithelium. During this time, there is a maintenance phase of the viral life cycle where the genome copy number is maintained at 20–50 copies per cell. In the upper layers of the infected epithelium there is a switch in replication to an activation mode that amplifies the viral genome copy number per cell. The control of this switch to amplification is not understood. The structural L1 and L2 proteins are made at this stage of the viral life cycle, and the viral genomes are encapsulated by these proteins [23]. The viral particles then egress from the epithelium; the viral life cycle therefore requires the differentiation of the host epithelium.

## 2. Human Papillomavirus Replication and the DNA Damage Response (DDR)

**Figure 1:**
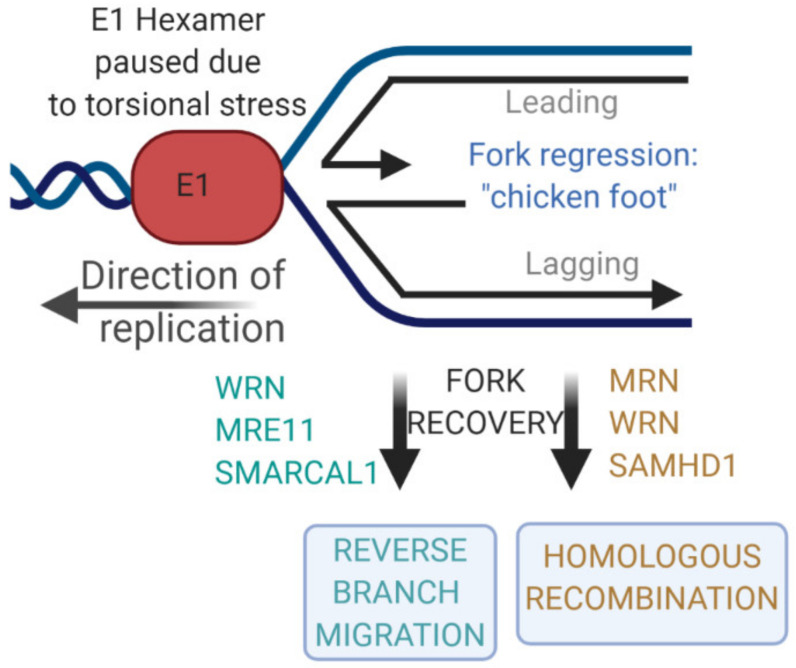
The E1 helicase pauses due to torsional stress on the viral genome undergoing replication. During replication this potentially causes fork regression, generating a chicken foot structure. This structure can be resolved without introducing a double-strand DNA break via reverse branch migration or homologous recombination. Some of the factors involved in these processes are highlighted. Please see Section 5 below for a more detailed description of the role of WRN (Werner helicase) in regulation of E1–E2 DNA replication.

HPV infection activates the DNA damage response (DDR), which is required for the amplification stage of the HPV life cycle [24]. There are several contributory factors regulating the DDR in HPV-positive cells that allow for the proliferation of the infected cell with an active DDR. External agents that induce the DDR ordinarily induce a cell cycle arrest that allows for the recognition and repair of the resulting DNA damage [25]. It is likely that the process of HPV replication per se activates the DDR [26,27,28,29,30]. Following infection, the rapid increase of the viral genome copy number to 20–50 copies per cell likely results in torsional stress on the viral genome. Replication forks “catching up” with each other could generate this torsional stress. Such stress can induce a chicken-foot structure due to fork regression (Figure 1) [31,32,33]. Two mechanisms resolve these structures without inducing a DNA double-strand break (DSB). First, reverse branch migration resolves the chicken foot, which involves a host of cellular factors and exonuclease digestion of the structure followed by the fork continuing to replicate the DNA in the original direction [33,34,35,36,37]. The second mechanism that resolves the chicken foot structure and promotes replication restart is homologous recombination (HR), a process that may occur without a DNA DSB from single-stranded DNA (ssDNA) generated at the reversed forks [38]. HPV replication uses HR, and a host of HR factors interact with HPV genomes [39,40,41,42,43,44,45,46,47]. The paused forks trigger the DDR via increased stretches of ssDNA that activate ATR and the recruitment of the MRE11-RAD50-NBS1 (MRN) complex that mediates HR and activates ATM; HPV-replicating DNA recruits MRN components [24,40,41,42,48,49,50]. This activation of the DDR is prevented from inducing a cell cycle arrest in the infected cells by the action of the E6 and E7 viral oncogenes, which disrupt the interaction of DDR proteins with host chromatin [51]. In HPV16 genomes that lack E6 and E7 expression (via introduction of stop codons), we demonstrated that the DDR is no different from that observed in wild-type HPV16 genome cells, but that the cells grow more slowly [52]. This result demonstrated the potential of the process of viral replication to activate the DDR in the absence of E6 and E7, and that E6 and E7 are important for promoting cell proliferation in the presence of the DDR induced by active replication. 

The ongoing viral replication in the presence of an active DDR presents an opportunity for studying DNA replication and repair in the presence of the DDR. In this review, we describe assays used to investigate the levels and fidelity of HPV16 replication. We discuss how these assays can be used to study lesions on DNA and how it can be used to study whether host factors play a role in promoting high fidelity replication via resolution of regressed replication forks. The purpose of this review is to stimulate the idea that this system can be more widely used to study DNA lesions and DDR proteins during replication. 

## 3. HPV16 DNA Replication Assays

The study of individual HPV proteins and processes is difficult due to the complicated viral life cycle; therefore, a reductionist approach is often taken for such studies. To do this for replication assays, E1 and E2 expression plasmids are co-transfected with a plasmid containing the viral origin of replication from the LCR (pOri) [53,54]. C33a cells, a cervical cancer cell line that has no HPV sequences present, are often used for such studies. Two to three days following transfection, low molecular weight is harvested from the transfected cells. The DNA is then treated with the restriction enzyme Dpn1, which cuts only the input plasmid prepared from bacteria. Dpn1 is a 4bp cutter that recognizes methylated sites; such methylation of DNA occurs in bacteria but not in mammalian cells. Traditionally, this DNA is then subjected to Southern blotting to separate the DNA and probe with a representative pOri plasmid. While qualitative, Southern blots have a lack of sensitivity when it comes to determining differences between samples. To overcome this, we developed a real-time qPCR assay for the replicated DNA [53,54]. There are many advantages to this protocol over Southern blotting; it is less toxic, quicker, and orders of magnitude more sensitive. In addition, Mbo1 digest of the harvested DNA digests the replicated DNA; Mbo1 recognizes the same 4bp sequence as Dpn1 that is non-methylated. Therefore, a combination of Dpn1 and Mbo1 digests allows quantitation of replication relative to the input DNA in the cell. Detailed technical descriptions are provided in [53,54]. The only additional step in this assay versus Southern blotting is to carry out an exonuclease III digestion step (Exo III) that digests the Dpn1/Mbo1 cut DNA and reduces the background in the PCR reaction. 

**Figure 2:**
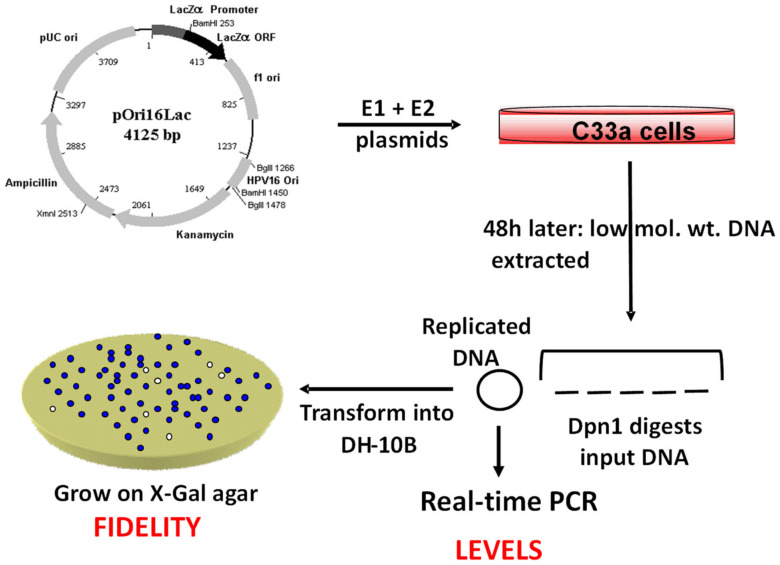
Measuring E1–E2 replication levels and fidelity. pOriLac was transfected along with E1 and E2 expression plasmids into C33a (cervical cancer cell line) cells. After 48 h, low molecular weight DNA was harvested and digested with Dpn1. The DNA was transformed into DH-10B and plated on X-gal agar. The mutation frequency was determined by the number of white colonies. The Dpn1 DNA was treated with Exo1 and real-time qPCR carried out to determine the levels of replication.

As well as measuring the quantity of DNA replication, this assay also offers the opportunity to develop fidelity of replication determination. To investigate fidelity, we incorporated the bacterial lacZ gene into the pOri plasmid (pOriLac) [55]. This plasmid can be transiently replicated by E1–E2 co-expression and low molecular weight DNA harvested and digested with Dpn1. The isolated replicated plasmids are transformed into bacteria, which are then plated onto agar containing a selectable marker along with X-gal. If the lacZ plasmid is intact, then blue colonies will arise; if the lacZ gene is mutated, then there will be white colonies. The increase in white colonies represents increased mutation frequency. Figure 2 details this protocol. It also has the advantage that the levels of DNA can be determined using our PCR approach. In this way, both the levels and fidelity of replication can be determined.

## 4. Using the HPV16 Replication Assay to Study Lesions on Replicating DNA

To learn more about the mechanisms of HPV16 E1–E2 (HPV16 from now on) replication and the host polymerases it uses, we investigated the ability of E1–E2 to use polη [55]. At the same time, these studies demonstrated the utility of the E1–E2 replication assay to study lesions introduced into pOriLac. To do this, we used Xeroderma Pigmentosum (XP) cell lines [56]. Patients with XP have a sensitivity to UV light and an increased incidence of skin cancer; they have a variety of lesions that prevent correct nucleotide excision repair (NER) in response to pyrimidine dimers induced on DNA exposed to UV radiation [57]. XPA patients have a mutation in the XPA gene; the XPA protein is involved in recognition of the DNA damage induced by UV and is required for efficient NER repair of the lesion [58]. Some XP variant (XPV) cells have a lesion in polη; polη is required for high-fidelity replication past a pyrimidine dimer and is therefore a replication and repair lesion [59]. We UVC irradiated pOriLac (Figure 2) and transfected it into a variety of cell lines including XP12 (a XPA cell line), XP30 (a XPV cell line) and XP30polη (XP30 with reconstituted polη expression). In all cell lines tested there was a large increase in mutation frequency following UVC treatment of pOriLac. In XP30polη, there was a highly significant decrease in mutation frequency compared with that observed in XP30 cells [55]. These results demonstrated that E1–E2 recruits polη during DNA replication. They also demonstrate that lesions introduced into pOriLac can be monitored *in vivo*. While this was done with a global UVC radiation of pOriLacZ, it is possible to introduce more targeted DNA lesions or roadblocks into the lacZ gene and determine whether the introduced lesion generates mutations in vivo in a variety of different genetic backgrounds.

## 5. Using the HPV16 Replication Assay to Study Lesions in Host DNA Replication and Repair Proteins

This section describes our investigation of host factors involved in regulating E1–E2 replication. We will demonstrate that our assay can measure the contribution of cellular factors to the maintenance of high-fidelity DNA replication. A reduction in the fidelity of replication following manipulation of host factors indicates that these factors are involved in a correct fork recovery possibly through reverse branch migration and/or HR (Figure 1). 

To identify host factors involved in E1–E2 replication, we carried out a yeast two-hybrid screen with E2, which identified TopBP1 as an interactor [60]. TopBP1 is a scaffold protein containing 9 BRCT (BRCA1 carboxyl terminus) domains and is involved in many aspects of nucleic acid metabolism, including DNA replication and repair [61]. Studies from several groups, including ours, have indicated that TopBP1 is important in HPV replication [43,44,45,48,62,63]. TopBP1 is an essential protein; therefore we sought to identify host enzymes that regulate TopBP1 as potential modulators of E1–E2 replication. The class III deacetylase SIRT1 is such an enzyme; it deacetylates TopBP1 and regulates its function in DNA replication [64,65]. We demonstrated that SIRT1 regulates E1–E2 replication; as well as binding to TopBP1, it also binds to E1 and E2 proteins and is part of the E1–E2 replication complex (as defined by chromatin immunoprecipitation (ChIP)) [47]. CRISPR/Cas9 editing of SIRT1 from C33a cells resulted in E1–E2 replication phenotypes. First, replication levels increased, at least in part due to increased acetylation and stability of E2. Second, the fidelity of replication was reduced in the absence of SIRT1 (as measured using our blue/white assay) [46,47]. To investigate the reason for the reduced fidelity of replication in the absence of SIRT1, we investigated changes in the recruitment of SIRT1 DNA replication and repair substrates to E1–E2-replicating DNA. Recruitment was measured using ChIP. We first checked TopBP1 levels, but recruitment of this protein was not changed in the absence of SIRT1. We therefore investigated other candidate proteins that are SIRT1 substrates and known to be involved in DNA damage repair and replication. Once such protein was the Werner helicase (WRN). The WRN protein is mutated in patients with Werner syndrome; sufferers have symptoms that include progeria and an increased incidence of cancer [66]. WRN encodes a 3′ to 5′ helicase and 3′ to 5′ exonuclease activity and has been implicated in many aspects of DNA damage and repair [67,68]. In replication and repair, the role of WRN is to assist with the maintenance and recovery of stalled replication forks (see Figure 1) [69,70,71,72,73]. In the absence of SIRT1, there is an increase in WRN acetylation [74,75]. In the absence of SIRT1 we observed an elevated level of WRN in C33a cells that was hyperacetylated; the acetylation of lysines may prevent the ubiquitination and proteasomal targeting of these residues [46]. However, the acetylated WRN could not interact with the E1–E2 replicating DNA, perhaps due to the increased negative charge following acetylation. We next CRISPR/Cas9 edited WRN from C33a cells and carried out replication assays. The results demonstrated an increased level of replication with a lower fidelity; this is a similar phenotype to that of SIRT1 knockout. 

**Figure 3:**
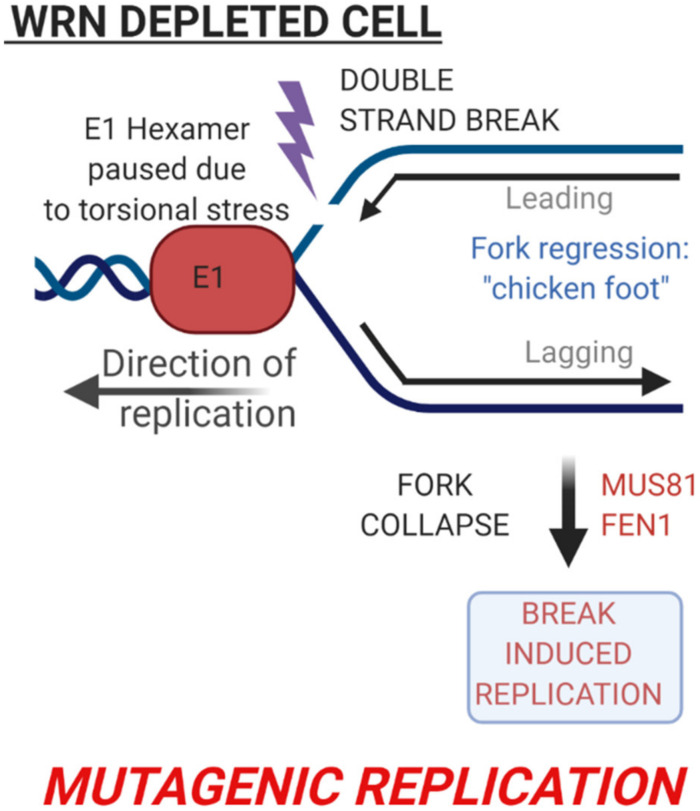
Mutagenic replication in the absence of WRN is potentially driven by break-induced replication. WRN depletion results in excess recruitment of MUS81 to E1–E2-replicating DNA. The endonuclease function of MUS81 cleaves the stalled fork, resulting in break-induced replication of the DNA, a mutagenic process.

Our assays demonstrated mutagenic replication following the elimination of host DNA replication and repair factors. Knockdown of TopBP1 also resulted in increased mutagenic E1–E2 replication [76]. What is the explanation for the increased replication levels and mutagenesis? Figure 1 highlights that WRN is involved in regulating both reverse branch migration as well as HR repair of stalled replication forks. The deletion of WRN would therefore reduce the efficiency or eliminate these functions; resolution of chicken foot structures via reverse branch migration involving WRN delays replication progression [72,77]. This would explain the increased levels of replication in the absence of WRN. Under these circumstances, E1–E2 replication would not be able to resolve the chicken foot structure. To continue with replication, one option is to carry out break-induced replication (BIR) [78,79,80]. Using this process, a DSB is introduced into the stalled fork, allowing the restart of replication from the DSB. BIR is more mutagenic than either reverse branch migration or HR resolution of the paused fork. In the absence of WRN, MUS81 is a candidate endonuclease that would introduce such a break [77,81,82]. Figure 3 summarizes BIR and the factors that may be involved in this process. In support of the switch from reverse branch migration and HR to BIR in the absence of WRN, more recently we demonstrated a significant increase of MUS81 recruitment to E1–E2-replicating DNA in the absence of WRN, as determined by ChIP; the FEN1 endonuclease also has enhanced recruitment in the absence of WRN (Das, Morgan, and Morgan, unpublished). Therefore, the HPV system has characterized a pathway already known to exist in the repair of replicating mammalian DNA. This demonstrates the utility of the system to provide a relatively quick dissection of replication and repair factors and their contribution to the control of the rates and fidelity of DNA replication under DNA damage conditions.

## 6. Summary

This short review highlights the use of E1–E2 replication assays for studying DNA lesions and DDR factors in mammalian cells. Another feature of this system is that we are easily, and inexpensively, able to determine the types of mutations that are occurring. If we sequence the lacZ gene from pOriLac plasmids isolated from the white colonies generated following replication, we can determine the precise genetic lesions that occur. Using restriction digest of the plasmid, we can easily identify large-scale rearrangements that have occurred. For example, in XP cell lines, the treatment of pOriLac with UVC prior to replication results in a dramatic and significant increase in the number of rearranged plasmids [55]. This is due to the collapse of the replication forks during the replication across pyrimidine dimers and a likely switch to break-induced replication that has enhanced mutagenesis. Rather than CRISPR/Cas9 edit cells, it is also possible to co-transfect a shRNA plasmid targeting host factors that disrupt replication foci and induce mutagenic replication. This was done with TopBP1 [76]. This allows a quick screening of many factors for their control of replication and repair in mammalian cells. In addition, E1–E2 replication persists when external DNA-damaging agents arrest host cell replication, allowing the study of replication mutations in the presence of external agents [76,83]. This system also allows for multiple targeting of cellular factors via co-transfection of multiple shRNA plasmids, or co-transfection into cells that have been CRISPR/Cas9 edited. An advantage of the CRISPR approach is that wild-type and mutant plasmids can complement the lesion in transient assays. For example, co-transfection of wild-type WRN plasmid in the replication assay in WRN CRISPR edited cells restores wild-type replication phenotypes [46]. Therefore, enzymatic and structural functions of host factors controlling replication and repair (WRN, for example) can quickly and easily be determined. Recruitment of fork remodeling, or HR, factors can be investigated at specific sequences in the replicating viral DNA using ChIP, thus uncovering mutual and spatial relationships between those factors. Overall, this system allows for the discovery and characterization of host factors that regulate the replication and repair of wild-type and damaged DNA templates. Finally, there is a host of epigenetic alterations that occur on HPV DNA during viral replication, therefore studying the consequences of the disruption of epigenetic regulators is another facet of this assay that could be exploited [84].
